# Fish and Shellfish-Derived Anti-Inflammatory Protein Products: Properties and Mechanisms

**DOI:** 10.3390/molecules26113225

**Published:** 2021-05-27

**Authors:** David C. Kemp, Jung Yeon Kwon

**Affiliations:** 1 Department of Food Science and Technology, Oregon State University, Corvallis, OR 97331, USA; David.c.kemp@oregonstate.edu; 2 Seafood Research and Education Center, Oregon State University, Astoria, OR 97103, USA

**Keywords:** seafood byproduct, marine, bioactive, hydrolysate, peptides, inflammation, anti-inflammatory, antioxidant

## Abstract

The interest in utilizing food-derived compounds therapeutically has been rising. With the growing prevalence of systematic chronic inflammation (SCI), efforts to find treatments that do not result in the side effects of current anti-inflammatory drugs are underway. Bioactive peptides (BAPs) are a particularly promising class of compounds for the treatment of SCI, and the abundance of high-quality seafood processing byproducts (SPB) makes it a favorable material to derive anti-inflammatory BAPs. Recent research into the structural properties of anti-inflammatory BAPs has found a few key tendencies including they tend to be short and of low molecular weight (LMW), have an overall positive charge, contain hydrophobic amino acids (AAs), and be rich in radical scavenging AAs. SPB-derived anti-inflammatory BAPs have been observed to work via inhibition of the NF-κB and MAPK pathways by disrupting the phosphorylation of IκBα and one or more kinases (ERK, JNK, and p38), respectively. Radical scavenging capacity has also been shown to play a significant role in the efficacy of SPB-derived anti-inflammatory BAPs. To determine if SPB-derived BAPs can serve as an effective treatment for SCI it will be important to understand their properties and mechanisms of action, and this review highlights such findings in recent research.

## 1. Introduction

Food has been used as a therapeutic treatment of diseases for millennia, however the discourse surrounding the role that food and food-derived compounds play in human health has been reinvigorated of late [1]. Recent studies have shown that numerous food processing byproducts can be minimally processed and utilized as value-added products, and in doing so eliminate waste, improve human health, protect the environment, and introduce new profit streams into the global economy [2,3,4,5].

Systemic chronic inflammation (SCI) is one of the greatest challenges to human health and wellbeing in the 21st century [6]. SCI promotes a plethora of negative health outcomes, and while there are a number of treatments, they tend to present negative side effects when utilized long-term [7]. This has driven a push for gentler methods to mitigate SCI [8]. Food processing byproducts are abundant, inexpensive, and contain a variety of compounds that may offer an innovative approach to the treatment of SCI [2,9]. 

Seafood processing byproducts (SPB) are an ideal candidate to derive useful biomolecules and have already garnered attention in the fields of nutrition, medicine, and cosmetics [10,11]. A large portion of harvested marine resources currently go to waste [12]. Around 60–70% of initial fish and shellfish weight is considered byproduct, and is utilized in low-value applications or discarded outright [13,14,15]. SPB consists of heads, frames, skin, shells, and viscera, much of which contain high-quality nutrients, including lipids, minerals, and protein [16]. A promising method for SPB utilization is the chemical, microbial, or enzymatic hydrolysis of byproduct into protein hydrolysates containing bioactive peptides [17]. Bioactive protein hydrolysates (BPHs) can be utilized outright, or be subdivided into highly bioactive fractions and bioactive peptides (BAPs) [18,19,20].

BAPs are small, liberated protein fragments, around 2-20 amino acid (AA) bases long. The small size and exposed AA side chains of BAPs enable ready interaction with other molecules [15,21,22]. BAPs are intriguing for the treatment of SCI, because they are readily processed by body, and may sidestep the undesirable side effects of current treatments [21]. As with any bioactive compound, peptides present a measure of risk including the potential to stimulate allergic reactions in some individuals, however with caution BAPs could show to be a viable treatment for SCI [23]. Therefore, fish- and shellfish-derived protein products are an important area of research in the effort to treat inflammation both chronic and acute, and to mitigate damage from inflammation-related diseases. Marine-derived peptides have previously been found to possess anti-oxidant, anti-tumor, anti-hypertensive, angiotensin-converting-enzyme (ACE) inhibitory, immunomodulatory, antidiabetic, antimicrobial, anticoagulant, and anti-inflammatory potential [1,24].

The properties of anti-inflammatory protein products and the mechanisms by which they act are intrinsically linked. A better understanding of how BAPs structural properties and mechanisms are connected will be required to harness their potential in the coming years. This review provides a resource in which properties and mechanisms of fish- and shellfish-derived anti-inflammatory protein products are discussed based on the recent evidence. Previous work by Chakrabarti et al., Bechaux et al., Venkatraman et al., Cal et al., Chalamaiah et al., Olsen et al., Daliri et al., La Manna et al., Zamora-Sillero et al., Nongonierma et al., Urakova et al., Guha et al., and Zhu et al. have expounded on aspects of marine-derived bioactive compounds [3,5,10,13,25,26,27,28,29,30,31,32,33,34]. This review builds upon these previous efforts and will update and expand on such works. 

## 2. Chronic Inflammation

Inflammation is an important biological mechanism that promotes healing and resolution in cases of infection and injury [34]. Furthermore, inflammation participates in homeostasis, cellular propagation, immune function, and generally resolves promptly in healthy individuals [35]. However, a variety of factors including infection, obesity, irregular sleep patterns, inactivity, metabolic dysfunction, exposure to toxins, tissue damage, and psychological stress can contribute to the propagation of inflammation over long periods of time [6], leading to SCI. SCI is implicated in conditions as diverse as type 2 diabetes, neurodegenerative disease, autoimmune disease, arthritis, atherosclerosis and other cardiovascular diseases, asthma, autoimmune, intestinal dysfunction, muscular atrophy, psoriasis, and it has been estimated that 20% of human cancers are associated with SCI [7,29,32,36,37].

Pancreatic cancer, for instance, can be initiated by DNA damage resulting from inflammation and the associated increase in reactive oxygen and nitrogen species [38]. SCI promotes muscle wasting, via nuclear factor-κB (NF-κB) activation and subsequent upregulation of muscle ubiquitin ligase (MuRF-1). Cytokines like tumor necrosis factor-α (TNF-α) and interleukin-6 (IL-6) are two of the most important bioindicators of muscle wasting [26]. Mechanical damage to skeletal joints can instigate an inflammatory response via the NF-κB pathway, which results in vascular leaks and perpetuates the cycle of SCI, and over time culminates in osteoarthritis [39].

NSAIDs are a common treatment for inflammation, both chronic and acute. While NSAIDs are an invaluable tool in combating inflammation, problems arise when used to treat chronic inflammation over long periods of time, the risk of associated gastric bleeding, ulceration, renal failure, and a myriad of other side effects can occur [24,25,40]. Anti-inflammatory BAPs have been proposed as a gentler alternative to NSAIDs [29,41]. BAPs are easily produced from underutilized materials including SPB, and if effective anti-inflammatory peptides can be characterized and established as safe for human use, they may be a powerful tool for promoting health and wellbeing, by mitigating the impact of chronic inflammation and related diseases.

## 3. Fish and Shellfish-Derived Anti-Inflammatory Protein Products: Properties and Mechanisms 

### 3.1. Properties

**Figure 1:**
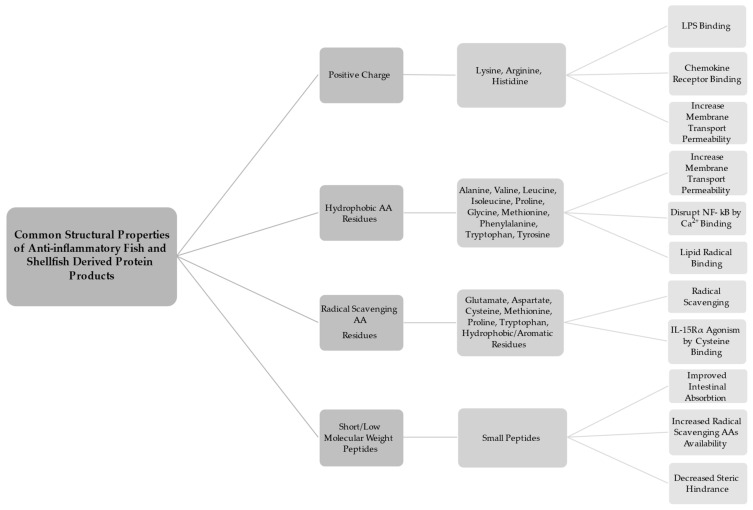
Common structural properties of anti-inflammatory fish- and shellfish-derived protein products.

A number of broad trends have been observed in the structure of anti-inflammatory peptides. Anti-inflammatory BAPs tend to be positively charged, have hydrophobic AA residues, be rich in antioxidative AAs, and be short, of low molecular weight (LMW) peptides (Figure 1) [34]. N- and C-terminal ends are of particular importance in BAPs [42]. There are notable exceptions, and some protein products do not adhere to one or more of the described trends, yet they still exhibit anti-inflammatory activity in experimental models. This may be partially attributable to the difference in peptides whose anti-inflammatory capacity is due to primary structure (AA sequence), and those that exhibit bioactivity due to secondary and or tertiary structure (peptide folding via hydrogen bonding and sidechain interaction) [34]. The following explores the trends observed in recent as to the structural properties of anti-inflammatory protein products.

Anti-inflammatory peptides tend to have an overall positive charge, and contain an abundance of positively charged AAs such as arginine, lysine, and histidine, particularly in the N- and/or C-terminal positions [43,44,45,46]. A study by Durand et al. [43] utilized electrodialysis to recover two cationic, and two anionic peptide fractions from herring milt hydrolysate. The cationic fractions showed significantly higher anti-inflammatory activity in lipopolysaccharide (LPS)-induced J774 mice macrophage cells than their anionic counterparts. One proposed reason for the observed activity is that cationic peptides can bind with LPS to stymie LPS-specific-stimulated inflammatory response [46]. It has also been suggested that positively charged AA residues may cause peptides to activate chemokine receptors thus exerting an anti-inflammatory effect [43,47]. Studies from Saisavoey et al. and Sangtanoo et al. point out that fish- and shellfish-derived protein products contain an abundance of cationic AAs [43,45]. Therefore, SPBs are ideal materials in which to search for positively charged peptides. It should be noted that some anti-inflammatory BAPs contain negatively charged AAs as well. Glutamine and arginine are common residues in anti-inflammatory peptides [48]. The presence of charged AAs may help to increase intestinal absorption. A study by Ding et al. [49] found that basic and acidic AAs at the N- and C- terminal ends of tetra- and pentapeptides increases transport permeability of across Caco-2 cell monolayers. A study by Gao et al. [46] showed that negatively charged peptides exhibited anti-inflammatory activity. While it is most common that anti-inflammatory BAPs have an overall positive charge the property of negative charge may improve anti-inflammatory potential as well.

Hydrophobic AA residues such as phenylalanine, leucine, tyrosine, glycine, and tryptophan, are often found in anti-inflammatory protein products [50]. This is especially true at the N-terminals [45,46,51]. A number of mechanisms have been proposed to explain this tendency. Ding et al. [49] showed that transport across a Caco-2 cell monolayer was increased in tetrapeptides with hydrophobic and sulfur-containing residues at the N-terminal (leucine, proline, isoleucine, cysteine, methionine, and valine), valine at the C-terminal, and pentapeptides with tyrosine at N- and C-terminals. Furthermore, the ability of highly hydrophobic peptides to interact with cell membranes has been hypothesized to disrupt inflammatory pathway cascades [46]. It has been suggested that elevated levels of glycine in peptides and hydrolysates can increase anti-inflammatory potential [52]. Glycine and small glycine-containing peptides have a high affinity for calcium binding [53], thus potentially disrupting Ca^2+^ signaling, which plays a central role in NF-κB signaling and cytokine production [54]. Glycine has been shown to have anti-inflammatory effects both in vitro and in vivo; this is potentially the result of glycine impacting intracellular Ca^2+^ levels by activation of glycine-gated chloride channels [55]. Glycine-rich peptides may be more likely to exhibit anti-inflammatory properties [56]. Furthermore, Sangtanoo et al. [45] note that hydrophobic AAs are attracted to lipid molecules and by extension linoleic acid, therefore they could potentially act as scavengers of lipid-derived radicals. This ties in with the next major property of anti-inflammatory fish and shellfish protein products which is that they are often rich in anti-oxidative AA residues.

Protein products that exhibit anti-inflammatory activity are often rich in radical scavenging AAs [57]. Glutamate and aspartate act as electron donors, and thus exhibit antioxidant capacity [44,46]. Additionally, it has been suggested that AAs containing radical scavenging side chains (including hydrophobic and aromatic AAs) may act to relieve oxidative stress and therefore offer protection from reactive oxygen species (ROS)-induced inflammation [15]. Several fish and shellfish studies including those by Durand et al., Giannetto et al., Saisavoey et al., and Sangtanoo et al. [15,43,44,45] noted high proline concentrations in their protein products. Proline has been indicated as an important AA in antioxidant peptides [43,51], and has been shown to ameliorate some of the negative impacts of LPS stimulation in rat cortex and cerebellum [58]. It should also be noted that warm water fish collagen generally contains more proline than that of cold-water fish [59]. In a study by Kaul et al. in vitro evidence suggests that L-proline acts as a radical scavenger [60]. There is evidence that cysteine may act as a radical scavenger as well [44,51,61,62], and cysteine has been implicated as an AA residue abundant in numerous anti-inflammatory BAPs [48]. One potential mechanism of cysteine’s anti-inflammatory activity is IL-15Rα binding and agonism [29]. A study by Peng et al. [62] found that hydrolysates high in radical scavenging peptides including cysteine, methionine, and tryptophan ameliorated the effects of UVB radiation in mice models by increasing enzyme activity of superoxide dismutase (SOD), and glutathione peroxidase (Gpx), while decreasing levels of malondialdehyde, IL-1β, IL-6, TNF-α, inducible nitric oxide synthase (iNOS), and cyclooxygenase-2 (COX-2) in mice models when applied topically. 

Anti-inflammatory protein products are often short, LMW peptides [43,52,63]. This is fortuitous because small peptides are more readily absorbed by the body, and this allows the possibility that the anti-inflammatory peptides may be utilized as orally administered therapeutics [3,64]. LMW protein products are more likely to cross the intestinal barrier intact, thus increasing the possibility that they can exert anti-inflammatory action in the body [34,48,65]. It has been shown by Ding et al. [49] that even certain tetra- and pentapeptides were able to cross a monolayer of Caco-2 cells. In the study by Ngamsuk et al. [66] <3 kDa hydrolysate fractions from rice milk showed the highest absorbability. Furthermore, it has been noted that small peptide size limits peptide folding and secondary/tertiary structure formation, which results in more radical scavenging AAs (especially those with hydrophobic side chains) being left exposed and able to scavenge radical species [67]. A number of longer peptides have been found to exhibit anti-inflammatory properties both in vivo and in vitro. For instance, the dairy-derived glycomacropeptide (GMP) (~7.5 kDa) and soy-derived lunasin and two more lunasin-like peptides (5, 8, and 14 kDa respectively) have shown robust anti-inflammatory activity, but would not be considered to be small peptides [34,65,68]. Furthermore, a study by Kim et al. [61] found that a blue mussel-derived, >5 kDa hydrolysate fraction exhibited higher anti-inflammatory bioactive properties than lower molecular weight 1–5, and <1 kDa fractions. The anti-inflammatory activity of larger peptides such as these is possibly due to secondary and tertiary structure of the molecules. This phenomenon has been observed with lunasin in an experiment that further digested the peptide into four fragments, none of which exhibited any anti-inflammatory activity [34,69]. Larger exceptions notwithstanding, it has been suggested that LMW peptides generally show higher levels of bioactivity because the small size minimizes steric hindrance and allows peptides access to more active sites on proteins [59]. LMW fish- and shellfish-derived protein products were reported in studies from Ahn et al., Gao et al., Saisavoey et al., Siregar et al., and Subhan et al. [44,59,63,65,70]. Studies by Joshi et al., Joshi and Nazeer, Kim et al., Narayanasamy et al. [24,48,56,61] found bioactive products in fractions >5 kDa. The study by Durand et al., 2020 [43] found two anti-inflammatory BAPs, with one being >5 kDa and one being <5 kDa.

It may be noted that these categories cover a broad range of possible peptide structures. The picture of what exactly the mechanism of anti-inflammatory peptides is fuzzy at best as of yet. However, as the pace of research into bioactive peptides increases, and as in silico and artificial intelligence approaches to bioactive research become more widely utilized the observed trends progress understanding [36,71,72,73]. 

### 3.2. Mechanisms

**Figure 2:**
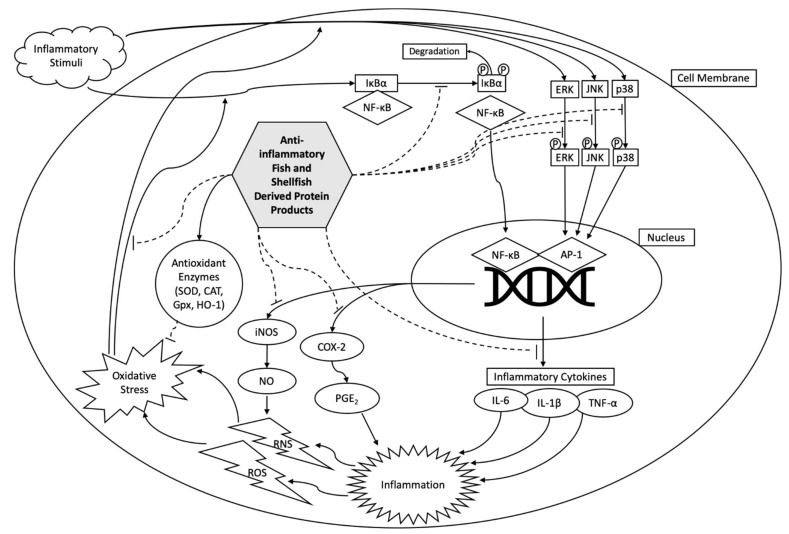
Potential anti-inflammatory mechanisms of action by fish- and shellfish-derived protein products.

Broadly, the main overarching mechanism of anti-inflammatory compounds is to decrease inflammatory chemokine and/or cytokine expression (Figure 2) [43]. NSAIDs work in this manner, however, numerous negative side effects are commonly reported as the result of long-term use [74]. Food-derived peptides and protein products with anti-inflammatory effects are generally safe and could potentially provide an alternative to NSAIDs with fewer undesirable side effects. Therefore, anti-inflammatory food-derived protein products including those derived from fish and shellfish are a promising field of research. NF-κB and MAPK pathways are the two main pathways explored when assessing the anti-inflammatory mechanisms of fish and shellfish-derived protein products. These two pathways are ubiquitous and easily evaluated via in vitro and in vivo models.

NF-κB is a family of inducible transcription factors that contribute to an immune and inflammatory response. NF-κB is a large biochemical pathway that stimulates the production of numerous cytokines and chemokines that can be easily measured via a multitude of methods, both in vivo and in vitro [7]. NF-κB induces expression of pro-inflammatory genes, upregulates pro-inflammatory cytokines such as IL-1, IL-2, IL-6, IL-8, IL-12, and TNF-α which induce an inflammatory response, cellular proliferation, and angiogenesis [75]. Chemokines such as MCP-1, IL-18, RANTES, MIP-2, CXCL1, CXCL10 are also mediated by NF-κB and can be indicators of modulated function. NF-κB mediates the inflammasome, can contribute to survival, activation, and differentiation of immune and T cells. Furthermore NF-κB is central to the induction of COX-2 and iNOS [54]. Deregulated NF-κB activation is a main contributor to chronic inflammatory diseases such as arthritis, atherosclerosis, multiple sclerosis, lupus, inflammatory bowel disease, diabetes, COPD, asthma, and various disorders [54,59,76]. 

The canonical pathway for NF-κB activation relies upon the phosphorylation and subsequent degradation of the subunit IκBα by IκB, which then allows for nuclear translocation of NF-κB [54,61,63]. A study from Kim et al. [61] found that a blue mussel hydrolysate fraction (>5 kDa) downregulated NF-κB by preventing phosphorylation of IκBα in RAW 264.7 murine macrophage (RAW 264.7) cells. Giannetto et al. and Gao et al. [15] reported that an anchovy protein hydrolysate and sturgeon muscle hydrolysate fraction respectively had anti-inflammatory action via the disruption of IκBα degradation, by direct measurement of cytosolic levels of phosphorylated IκBα in RAW 264.7 cells.

COX-2 stimulates the conversion of arachidonic acid into prostaglandins, and is the target of most NSAIDs [48]. Prostaglandin E2 (PGE2) is a notable product of COX-2 and is upregulated during inflammation. Therefore, inhibition of COX-2 and PGE2 are primary targets in the search for anti-inflammatory compounds with limited side effects. COX-2 and PGE2 inhibition has been found in numerous studies of fish and shellfish-derived protein products indicating that these products may be developed as useful alternatives for NSAIDs in the future [48]. For instance, studies from Kim et al. [61] and Ahn et al. [65] found that hydrolysates from blue mussel, and the peptide PAY from salmon pectoral fin respectively were found to inhibit COX-2 and PGE2 expression RAW 264.7 cells.

IL-1β and IL-6 are cytokines that are upregulated during inflammation. IL-1β deregulation is implicated in inflammation related pain as well as many of the numerous chronic inflammation-related diseases previously mentioned [77]. IL-6 upregulates production of several compounds related to chronic inflammatory including C-reactive protein and fibrinogen [78]. Therefore, measuring the expression of these cytokines is the main focus of many of the studies in this review. For instance a <1.5 kDa hydrolysate fraction from sturgeon muscle, and peptide PAY from salmon pectoral fin inhibited IL-1β and IL-6 expression in LPS-stimulated RAW 264.7 cells [63,65]. Furthermore, crude hydrolysates from anchovy viscera reduced IL-6, but not IL-1β in mice aorta tissue [15].

Activation of MAPK is another notable cellular process under inflammation. MAPK is a highly conserved pathway integral to numerous cellular functions including cell proliferation, differentiation, and inflammatory response [46,79]. MAPK activation is primarily reliant upon the phosphorylation of three protein kinases, namely, ERK, JNK, and p38. Anti-inflammatory peptides that work on the MAPK system rely upon the disruption of the phosphorylation of ERK, JNK, and p38. Each of these three kinases is phosphorylated in a three-step process that each include a tyrosine and threonine at the activation site [76]. Therefore, it has been proposed that peptides that can bind with any one of these active sites may disrupt the MAPK pathway [34]. The study from Gao et al. [46] showed that some protein products can interrupt JNK and p38 phosphorylation, while not significantly impacting the phosphorylation of ERK. Another study found that a pepsin hydrolysate fraction of <1.5 kDa from sturgeon protein disrupted phosphorylation of ERK, JNK, and p38 in RAW 264.7 cells [63].

TNF-α is cytokine produced by macrophage cells during inflammation and is both an activator of inflammatory pathways and a product of inflammation. TNF-α expression is regulated by both MAPK and NF-κB pathways. Research by Gao et al. [46] indicates that TNF-α downregulation relies on the disruption of phosphorylation of the ERK transcription factor. This research reports several peptides derived from sturgeon promoted a decrease in JNK and p38 phosphorylation as well as a decrease in IL-1β and IL-6, but notably no significant decrease in TNF-α secretion or ERK phosphorylation was observed. This suggests that TNF-α is regulated by both the NF-κB and MAPK pathways and has to do with specific disruption of the ERK transcription factor [46]. Hydrolysates from anchovy viscera, sturgeon muscle, blue mussel, threadfin bream and peptides from salmon bones, saltwater clams, and salmon pectoral fin have all been reported to downregulate TNF-α levels in LPS-stimulated RAW 264.7 cells (Table 1) [15,44,48,51,61,63,65]. The study from Giannetto et al. [15] reported TNF-α downregulation in mice aorta tissue in groups treated with diets supplemented with anchovy protein hydrolysates, and Joshi et al. [48] found TNF-α downregulation in LPS-stimulated zebrafish treated with peptide HKGQCC derived from saltwater clams.

Oxidative stress and inflammation are intimately connected. Reactive oxygen species and reactive nitrogen species (RNS) are ubiquitous in the body and regulate cellular function when at healthy levels [14,70]. However, oxidative stress plays a central role in chronic inflammation when dysregulated [80]. Excessive oxidative stress activates both the NF-κB and MAPK pathways, stimulating an inflammatory response, this in turn promotes the production of more reactive species and depletes cellular antioxidants thus perpetuating a cycle of inflammation [81]. This cycle has been implicated as one of the main drivers of chronic inflammation [9,51]. Oxidative stress can be mitigated by cellular production of enzymes that break down reactive molecules. Antioxidant enzymes such as SOD, catalase (CAT), Gpx, and heme oxygenase-1 (HO-1) [46]. Another approach to mitigating oxidative stress is reducing the production of reactive species to begin with. During inflammatory response, iNOS catalyzes the transformation of L-arginine into nitric oxide (NO) [56]. NO can be easily measured colorimetrically by the addition of Griess reagent, therefore NO production assays are often a starting point in anti-inflammatory compound studies [44]. Studies of milkfish scale collagen peptide fractions (<3 kDa), peptides IVPAS and FDKPVSPLL from herring milt, and peptide HKGQC from saltwater clams were all found to decrease NO production in LPS-stimulated RAW 264.7 cells [14,43,48].

**Table 1:** Described mechanisms of fish- and shellfish-derived protein products.

Protein Product	Source	Preparation Method	Model(s)	Mechanism(s)	Reference
Peptides: IVPAS, FDKPVSPLL	Herring Milt	From Hydrolysate Enzyme(s): mix (confidential)	Cellular: LPS-stimulated J774 mouse macrophage cells	Inhibition of iNOS activity, oxygen radical scavenging.	[43]
Peptide: EGLLGDVF	Green mussel	From Hydrolysate Enzyme(s): Alcalase	Cellular: LPS-stimulated RAW 264.7 mouse macrophage cells	Downregulation of iNOS and COX-2 protein expression.	[56]
Peptide: LGLGAAVL	Marine crab leg muscle	From Hydrolysate Enzyme(s): Trypsin	Cellular: LPS-stimulated RAW 264.7 mouse macrophage cells	Suppression of COX-2 expression.	[24]
Hydrolysate	Threadfin bream muscle protein	Hydrolysate fraction: Treated with ultrasound 300W, and Microwave 100W Enzyme(s): Alcalase	Chemical: ABTS and DPPH radical scavenging assays Cellular: H_2_O_2_-stimulated *RAW* 264.7 mouse macrophage cells	Radical scavenging. Upregulation of SOD and CAT activity. Downregulation of TNF-α and IL-1β.	[51]
Collagen Peptide Fraction: <3 kDa collagen peptide	Milkfish scales	Collagen extraction and ultrafiltration	Chemical: Lox-1 activity and nitric oxide radical production assays	Decrease in ROS and RNS activity via inhibition of lipoxygenase and NO	[14]
Hydrolysate: high molecular weight (>5 kDa) fraction	Blue mussel	From Hydrolysate: Enzyme: Pepsin	Cellular: LPS-stimulated RAW 264.7 mouse macrophage cells	Inhibition of iNOS and COX-2 gene expression and reduction in TNF-α, IL-6, and IL-1β. Inhibition of of NF-κB translocation by preventing phosphorylation of IκBα and inhibition of PGE2 secretion.	[61]
Peptide: YA	Oyster	From Hydrolysate Enzyme(s): Protamex, and Neutrase	Chemical: COX-2 and 5-LOX activity assays Cellular: IL-1β and TNF-α mRNA expression in EtOH induced Chang liver cells	Inhibition of COX-2 and 5-LO, and a reduction in IL-1β, TNF-α	[70]
Peptides: KIWHHTF, VHYAGTVDY, HLDDALRGQE	Sturgeon back muscle	From Hydrolysate Enzyme(s): Pepsin	Cellular: LPS-stimulated RAW 264.7 mouse macrophage cells	Downregulation of JNK and p38 phosphoylation. Decrease in IL-1β and IL-6. Upregulation of SOD activity.	[46]
Hydrolysates	Anchovy (*Engraulis encrasicolus*) viscera	From Hydrolysate Enzyme(s): Protamex, Flavourzyme, and Alcalase	Cellular: LPS-stimulated RAW 264.7 mouse macrophage cells in vivo: female six-month-old B6.129P2-ApoE−/− mice	Inhibition of COX-2 and the nuclear translocation of NF-κB by preventing phosphorylation of IκBα. Modulation of iNOS, MnSOD and HO-1 expression. Downregulation of TNF-α, IL-1α, and IL-6, and modulation of SOD, catalase (CAT), Glutathione peroxidase (Gpx), and Heme oxygenase in mice aorta and heart tissue.	[15]
Hydrolysate Fraction: <1.5 kDa	Sturgeon Muscle	From Hydrolysate Enzyme(s): Pepsin	Chemical: ABTS and DPPH radical scavenging assays Cellular: LPS-stimulated RAW 264.7 mouse macrophage cells	Radical scavenging. Inhibition of IL-1β, IL-6, TNF-α, and phosphrylation of MAPKs/IκBα	[63]
Peptides: SNKGGGRPN, PGVATAPTH, LLGLGLPPA	Salmon Bones	From Hydrolysate Enzyme(s): Papain	Cellular: LPS-stimulated RAW 264.7 mouse macrophage cells	Inhibition of NO, COX-2, IL-6, iNOS, and TNF-α mRNA	[44]
Peptide: HKGQCC	Saltwater clams (*Meretrix meretrix*)	From Hydrolysate Enzyme(s): Trypsin	Chemical: Albumin denaturation assay Cellular: Human red blood cell (HRBC) stabilization assay Cellular: LPS-stimulated RAW 264.7 mouse macrophage cells in vivo: 6–7 month old wild type zebrafish	Inhibition of NO, TNF-α, IL-1β, and COX-2 in LPS-stimulated RAW 264.7 cells Inhibition of TNF-α, IL-1β, iNOS, and COX-2 mRNA in LPS-stimulated Zebrafish	[48]
Peptide: PAY	Salmon pectoral fin	From Hydrolysate Enzyme: Pepsin	Cellular: LPS-stimulated RAW 264.7 mouse macrophage cells	Inhibition of NO/iNOS, PGE_2_/COX-2 pathways. Reduced TNF-α, IL-6, and IL-1β expression.	[65]
Hydrolysates	Sandfish (*Arctoscopus japonicus*) muscle (MHA) and roe (RHC)	From Hydrolysate Enzymes: Collupulin MG (RHC), and Alcalase (MHA)	Chemical: DPPH radical scavenging assay	Radical scavenging.	[82]

## 4. Conclusions

Chronic inflammation is one of the preeminent threats to the human health in the 21st century, in fact over 50% of deaths worldwide are attributable to diseases associated with chronic inflammation [6]. It is imperative to uncover more therapies for chronic inflammatory disease, that do not present the risks of long-term use of NSAIDs. Though most studies are still in preliminary stages and more research is required, anti-inflammatory protein products derived from fish and shellfish may have the potential to offer safe and effective alternatives to other more aggressive anti-inflammatory therapies for chronic inflammation in some cases [25]. Fish accounts for around 20% of the world’s animal protein intake [83], and nearly 60% of the total biomass of fish manufacturing is considered low value or waste, however this waste contains much of the total protein value of the fish [15]. Using even a small portion of this waste to generate anti-inflammatory protein products could help ameliorate the impact of chronic inflammation, reduce environmental strain, and generate new revenue streams [13]. Though not fully elucidated, it is clear that structural properties of anti-inflammatory fish- and shellfish-derived protein products are directly connected to function, and responsible for the mechanisms of action [28]. Therefore, it is important to consider properties and mechanisms of anti-inflammatory fish- and shellfish-derived products in tandem to improve the understanding and precision moving forward.

## Data Availability

Not Applicable.

## Abbreviations

AA	Amino Acid
BAP	Bioactive Peptide
BPH	Bioactive Protein Hydrolysate
CAT	Catalase
COX-2	Cyclooxygenase-2
Gpx	Glutathione Peroxidase
HO-1	Heme Oxygenase-1
iNOS	Inducible Nitric Oxide Synthase
kDa	Kilodalton
LMW	Low-Molecular Weight
LPS	Lipopolysaccharide
NSAID	Non-Steroidal Anti-inflammatory Drug
PGE2	Prostaglandin E2
RNS	Reactive Nitrogen Species
ROS	Reactive Oxygen Species
SCI	Systemic Chronic Inflammation
SOD	Superoxide Dismutase
SPB	Seafood Processing Byproduct

## References

[1] Venkatraman K.L., Mehta A. (2019). Health benefits and pharmacological effects of *Porphyra* species. Plant Foods Hum. Nutr..

[2] Torres-León C., Ramírez-Guzman N., Londoño-Hernandez L., Martinez-Medina G.A., Díaz-Herrera R., Navarro-Macias V., Alvarez-Pérez O.B., Picazo B., Villarreal-Vázquez M., Ascacio-Valdes J. (2018). Food waste and byproducts: An opportunity to minimize malnutrition and hunger in developing countries. Front. Sustain. Food Syst..

[3] Chakrabarti S., Guha S., Majumder K. (2018). Food-derived bioactive peptides in human health: Challenges and opportunities. Nutrients.

[4] Takayama K., Rentier C., Asari T., Nakamura A., Saga Y., Shimada T., Nirasawa K., Sasaki E., Muguruma K., Taguchi A. (2017). Development of potent myostatin inhibitory peptides through hydrophobic residue-directed structural modification. ACS Med. Chem. Lett..

[5] Bechaux J., Gatellier P., Le Page J.-F., Drillet Y., Sante-Lhoutellier V. (2019). A comprehensive review of bioactive peptides obtained from animal byproducts and their applications. Food Funct..

[6] Furman D., Campisi J., Verdin E., Carrera-Bastos P., Targ S., Franceschi C., Ferrucci L., Gilroy D.W., Fasano A., Miller G.W. (2019). Chronic inflammation in the etiology of disease across the life span. Nat. Med..

[7] Patil K.R., Mahajan U.B., Unger B.S., Goyal S.N., Belemkar S., Surana S.J., Ojha S., Patil C.R. (2019). Animal models of inflammation for screening of anti-inflammatory drugs: Implications for the discovery and development of phytopharmaceuticals. Int. J. Mol. Sci..

[8] Liu X., Lin Y.-J., Cheng Y. (2016). Complementary and alternative therapies for inflammatory diseases. Evid. Based Complement. Altern. Med..

[9] Ruijters E., Haenen G., Willemsen M., Weseler A., Bast A. (2016). Food-derived bioactives can protect the anti-inflammatory activity of cortisol with antioxidant-dependent and -independent mechanisms. Int. J. Mol. Sci..

[10] Venkatesan J., Anil S., Kim S.-K., Shim M. (2017). Marine fish proteins and peptides for cosmeceuticals: A review. Mar. Drugs.

[11] Gündüz H., Göztürk F., Hamzaçebi S., Akpınar M.D. (2018). The assessment of seafood processing waste. Aquat. Sci. Eng..

[12] Petrova I., Tolstorebrov I., Eikevik T.M. (2018). Production of fish protein hydrolysates step by step: Technological aspects, equipment used, major energy costs and methods of their minimizing. Int. Aquat. Res..

[13] Olsen R.L., Toppe J., Karunasagar I. (2014). Challenges and realistic opportunities in the use of by-products from processing of fish and shellfish. Trends Food Sci. Technol..

[14] Chen Y.-P., Liang C.-H., Wu H.-T., Pang H.-Y., Chen C., Wang G.-H., Chan L.-P. (2018). Antioxidant and anti-inflammatory capacities of collagen peptides from milkfish (*Chanos chanos*) scales. J. Food Sci. Technol..

[15] Giannetto A., Esposito E., Lanza M., Oliva S., Riolo K., di Pietro S., Abbate J.M., Briguglio G., Cassata G., Cicero L. (2020). Protein hydrolysates from anchovy (*Engraulis encrasicolus*) waste: In vitro and in vivo biological activities. Mar. Drugs.

[16] FAO (2016). Fish and Their By-Products.

[17] Surasani V.K.R. (2018). Acid and alkaline solubilization (PH Shift) process: A better approach for the utilization of fish processing waste and by-products. Environ. Sci. Pollut. Res..

[18] Coppola D., Lauritano C., Palma Esposito F., Riccio G., Rizzo C., de Pascale D. (2021). Fish waste: From problem to valuable resource. Mar. Drugs.

[19] Wan A.H.L., Davies S.J., Soler-Vila A., Fitzgerald R., Johnson M.P. (2019). Macroalgae as a sustainable aquafeed ingredient. Rev. Aquac..

[20] Rengasamy K.R., Mahomoodally M.F., Aumeeruddy M.Z., Zengin G., Xiao J., Kim D.H. (2020). Bioactive compounds in seaweeds: An overview of their biological properties and safety. Food Chem. Toxicol..

[21] Mora L., Aristoy M.-C., Toldrá F. (2019). Bioactive peptides. Encyclopedia of Food Chemistry.

[22] Chalamaiah M., Ulug S.K., Hong H., Wu J. (2019). Regulatory requirements of bioactive peptides (protein hydrolysates) from food proteins. J. Funct. Foods.

[23] Liu L., Li S., Zheng J., Bu T., He G., Wu J. (2020). Safety considerations on food protein-derived bioactive peptides. Trends Food Sci. Technol..

[24] Narayanasamy A., Balde A., Raghavender P., Shashanth D., Abraham J., Joshi I., Nazeer R.A. (2020). Isolation of marine crab (*Charybdis natator*) leg muscle peptide and its anti-inflammatory effects on macrophage cells. Biocatal. Agric. Biotechnol..

[25] Chakrabarti S., Jahandideh F., Wu J. (2014). Food-derived bioactive peptides on inflammation and oxidative stress. Biomed. Res. Int..

[26] Cal R., Davis H., Kerr A., Wall A., Molloy B., Chauhan S., Trajkovic S., Holyer I., Adelfio A., Khaldi N. (2020). Preclinical evaluation of a food-derived functional ingredient to address skeletal muscle atrophy. Nutrients.

[27] Chalamaiah M., Yu W., Wu J. (2018). Immunomodulatory and anticancer protein hydrolysates (peptides) from food proteins: A review. Food Chem..

[28] Daliri E., Oh D., Lee B. (2017). Bioactive peptides. Foods.

[29] La Manna S., di Natale C., Florio D., Marasco D. (2018). Peptides as therapeutic agents for inflammatory-related diseases. Int. J. Mol. Sci..

[30] Zamora-Sillero J., Gharsallaoui A., Prentice C. (2018). Peptides from fish by-product protein hydrolysates and its functional properties: An overview. Mar. Biotechnol..

[31] Nongonierma A.B., FitzGerald R.J. (2017). Strategies for the Discovery and Identification of Food Protein-Derived Biologically Active Peptides. Trends Food Sci. Technol..

[32] Zhu W., Ren L., Zhang L., Qiao Q., Farooq M.Z., Xu Q. (2020). The Potential of Food Protein-Derived Bioactive Peptides against Chronic Intestinal Inflammation. Mediat. Inflamm..

[33] Urakova I.N., Pozharitskaya O.N., Demchenko D.V., Shikov A.N., Makarov V.G. (2012). The biological activities of fish peptides and methods of their isolation. Russ. J. Mar. Biol..

[34] Guha S., Majumder K. (2019). Structural-features of food-derived bioactive peptides with anti-inflammatory activity: A brief review. J. Food Biochem..

[35] Calder P.C., Ahluwalia N., Albers R., Bosco N., Bourdet-Sicard R., Haller D., Holgate S.T., Jönsson L.S., Latulippe M.E., Marcos A. (2013). A consideration of biomarkers to be used for evaluation of inflammation in human nutritional studies. Br. J. Nutr..

[36] Khatun M.S., Hasan M.M., Kurata H. (2019). PreAIP: Computational prediction of anti-inflammatory peptides by integrating multiple complementary features. Front. Genet..

[37] Crusz S.M., Balkwill F.R. (2015). Inflammation and cancer: Advances and new agents. Nat. Rev. Clin. Oncol..

[38] Padoan A., Plebani M., Basso D. (2019). Inflammation and pancreatic cancer: Focus on metabolism, cytokines, and immunity. Int. J. Mol. Sci..

[39] Sokolove J., Lepus C.M. (2013). Role of inflammation in the pathogenesis of osteoarthritis: Latest findings and interpretations. Ther. Adv. Musculoskelet. Dis..

[40] Marcum Z.A., Hanlon J.T. (2010). Recognizing the risks of chronic nonsteroidal anti-inflammatory drug use in older adults. Ann. Longterm. Care.

[41] Majumder K., Mine Y., Wu J. (2016). The potential of food protein-derived anti-inflammatory peptides against various chronic inflammatory diseases. J. Sci. Food Agric..

[42] Selamassakul O., Laohakunjit N., Kerdchoechuen O., Yang L., Maier C.S. (2020). Bioactive peptides from brown rice protein hydrolyzed by bromelain: Relationship between biofunctional activities and flavor characteristics. J. Food Sci..

[43] Durand R., Pellerin G., Thibodeau J., Fraboulet E., Marette A., Bazinet L. (2020). Screening for metabolic syndrome application of a herring by-product hydrolysate after its separation by electrodialysis with ultrafiltration membrane and identification of novel anti-inflammatory peptides. Sep. Purif. Technol..

[44] Saisavoey T., Sangtanoo P., Reamtong O., Karnchanatat A. (2019). Free radical scavenging and anti-inflammatory potential of a protein hydrolysate derived from salmon bones on RAW 264.7 macrophage cells. J. Sci. Food Agric..

[45] Sangtanoo P., Srimongkol P., Saisavoey T., Reamtong O., Karnchanatat A. (2020). Anti-inflammatory action of two novel peptides derived from peanut worms (*Sipunculus nudus*) in lipopolysaccharide-induced RAW264.7 macrophages. Food Funct..

[46] Gao R., Shu W., Shen Y., Sun Q., Bai F., Wang J., Li D., Li Y., Jin W., Yuan L. (2020). Sturgeon protein-derived peptides exert anti-inflammatory effects in LPS-stimulated RAW264.7 macrophages via the MAPK pathway. J. Funct. Foods.

[47] Vogel H.J., Schibli D.J., Jing W., Lohmeier-Vogel E.M., Epand R.F., Epand R.M. (2002). Towards a structure-function analysis of bovine lactoferricin and related tryptophan- and arginine-containing peptides. Biochem. Cell Biol..

[48] Joshi I., Mohideen H.S., Nazeer R.A. (2021). A *Meretrix meretrix* visceral mass derived peptide inhibits lipopolysaccharide-stimulated responses in RAW264.7 cells and adult zebrafish model. Int. Immunopharmacol..

[49] Ding L., Wang L., Yu Z., Ma S., Du Z., Zhang T., Liu J. (2017). Importance of terminal amino acid residues to the transport of oligopeptides across the caco-2 cell monolayer. J. Agric. Food Chem..

[50] Yu W., Field C.J., Wu J. (2018). Purification and identification of anti-inflammatory peptides from spent hen muscle proteins hydrolysate. Food Chem..

[51] Li Z., Wang J., Zheng B., Guo Z. (2020). Impact of combined ultrasound-microwave treatment on structural and functional properties of golden threadfin bream (*Nemipterus virgatus*) myofibrillar proteins and hydrolysates. Ultrason. Sonochem..

[52] Joshi I., Sudhakar S., Nazeer R.A. (2016). Anti-inflammatory properties of bioactive peptide derived from gastropod influenced by enzymatic hydrolysis. Appl. Biochem. Biotechnol..

[53] Tang N., Skibsted L.H. (2016). Calcium binding to amino acids and small glycine peptides in aqueous solution: Toward peptide design for better calcium bioavailability. J. Agric. Food Chem..

[54] Liu T., Zhang L., Joo D., Sun S.-C. (2017). NF-ΚB signaling in inflammation. Signal Transduct. Target. Ther..

[55] Li X., Bradford B.U., Wheeler M.D., Stimpson S.A., Pink H.M., Brodie T.A., Schwab J.H., Thurman R.G. (2001). Dietary glycine prevents peptidoglycan polysaccharide-induced reactive arthritis in the rat: Role for glycine-gated chloride channel. Infect. Immun..

[56] Joshi I., Nazeer R.A. (2020). EGLLGDVF: A novel peptide from green mussel *Perna viridis* foot exerts stability and anti-inflammatory effects on LPS-stimulated RAW264.7 cells. Protein Pept. Lett..

[57] Dadar M., Shahali Y., Chakraborty S., Prasad M., Tahoori F., Tiwari R., Dhama K. (2019). Antiinflammatory peptides: Current knowledge and promising prospects. Inflamm. Res..

[58] Andrade V.S., Rojas D.B., de Andrade R.B., Kim T.D.H., Vizuete A.F., Zanatta Â., Wajner M., Gonçalves C.-A.S., Wannmacher C.M.D. (2017). A possible anti-inflammatory effect of proline in the brain cortex and cerebellum of rats. Mol. Neurobiol..

[59] Subhan F., Kang H.Y., Lim Y., Ikram M., Baek S.-Y., Jin S., Jeong Y.H., Kwak J.Y., Yoon S. (2017). Fish scale collagen peptides protect against CoCl 2 /TNF-α-induced cytotoxicity and inflammation via inhibition of ROS, MAPK, and NF-κB pathways in HaCaT cells. Oxid. Med. Cell. Longev..

[60] Kaul S., Sharma S.S., Mehta I.K. (2008). Free radical scavenging potential of L-proline: Evidence from in vitro assays. Amino Acids.

[61] Kim Y.-S., Ahn C.-B., Je J.-Y. (2016). Anti-inflammatory action of high molecular weight *Mytilus edulis* hydrolysates fraction in LPS-induced RAW264.7 macrophage via NF-ΚB and MAPK pathways. Food Chem..

[62] Peng Z., Chen B., Zheng Q., Zhu G., Cao W., Qin X., Zhang C. (2020). Ameliorative effects of peptides from the oyster (*Crassostrea hongkongensis*) protein hydrolysates against UVB-induced skin photodamage in mice. Mar. Drugs.

[63] Gao R., Shu W., Shen Y., Sun Q., Jin W., Li D., Li Y., Yuan L. (2021). Peptide fraction from sturgeon muscle by pepsin hydrolysis exerts anti-inflammatory effects in LPS-stimulated RAW264.7 macrophages via MAPK and NF-ΚB pathways. Food Sci. Hum. Wellness.

[64] Xiang X.-W., Zhou X.-L., Wang R., Shu C.-H., Zhou Y.-F., Ying X.-G., Zheng B. (2021). Protective effect of tuna bioactive peptide on dextran sulfate sodium-induced colitis in mice. Mar. Drugs.

[65] Ahn C.-B., Cho Y.-S., Je J.-Y. (2015). Purification and anti-inflammatory action of tripeptide from salmon pectoral fin byproduct protein hydrolysate. Food Chem..

[66] Ngamsuk S., Hsu J.-L., Huang T.-C., Suwannaporn P. (2020). Ultrasonication of milky stage rice milk with bioactive peptides from rice bran: Its bioactivities and absorption. Food Bioprocess Technol..

[67] Bamdad F., Bark S., Kwon C.H., Suh J.-W., Sunwoo H. (2017). Anti-inflammatory and antioxidant properties of peptides released from β-lactoglobulin by high hydrostatic pressure-assisted enzymatic hydrolysis. Molecules.

[68] Neelima R.S., Rajput Y.S., Mann B. (2013). Chemical and functional properties of glycomacropeptide (GMP) and its role in the detection of cheese whey adulteration in milk: A review. Dairy Sci. Technol..

[69] Hernández-Ledesma B., Hsieh C.-C., de Lumen B.O. (2009). Antioxidant and anti-inflammatory properties of cancer preventive peptide lunasin in RAW 264.7 macrophages. Biochem. Biophys. Res. Commun..

[70] Siregar A.S., Nyiramana M.M., Kim E.-J., Shin E.-J., Woo M.S., Kim J.-M., Kim J.H., Lee D.K., Hahm J.R., Kim H.J. (2020). Dipeptide YA is responsible for the positive effect of oyster hydrolysates on alcohol metabolism in single ethanol binge rodent models. Mar. Drugs.

[71] Kim J.H., Kim J.H., Sutikno L.A., Lee S.B., Jin D.H., Hong Y.K., Kim Y.S., Jin H.J. (2019). Identification of the minimum region of flatfish myostatin propeptide (Pep45-65) for myostatin inhibition and its potential to enhance muscle growth and performance in animals. PLoS ONE.

[72] Sanjeewa K.K.A., Nagahawatta D.P., Yang H.-W., Oh J.Y., Jayawardena T.U., Jeon Y.-J., de Zoysa M., Whang I., Ryu B. (2020). Octominin inhibits LPS-induced chemokine and pro-inflammatory cytokine secretion from RAW 264.7 macrophages via blocking TLRs/NF-ΚB signal transduction. Biomolecules.

[73] Farhadi T., Hashemian S.M. (2018). Computer-aided design of amino acid-based therapeutics: A review. Drug Des. Devel. Ther..

[74] Dinarello C.A. (2010). Anti-inflammatory agents: Present and future. Cell.

[75] Kany S., Vollrath J.T., Relja B. (2019). Cytokines in inflammatory disease. Int. J. Mol. Sci..

[76] Sabio G., Davis R.J. (2014). TNF and MAP kinase signalling pathways. Semin. Immunol..

[77] Ren K., Torres R. (2009). Role of interleukin-1β during pain and inflammation. Brain Res. Rev..

[78] Tanaka T., Narazaki M., Kishimoto T. (2014). IL-6 in inflammation, immunity, and disease. Cold Spring Harb. Perspect. Biol..

[79] Moens U., Kostenko S., Sveinbjørnsson B. (2013). The role of mitogen-activated protein kinase-activated protein kinases (MAPKAPKs) in inflammation. Genes.

[80] Lin Y., Jiang M., Chen W., Zhao T., Wei Y. (2019). Cancer and ER stress: Mutual crosstalk between autophagy, oxidative stress and inflammatory response. Biomed. Pharmacother..

[81] Vaziri N.D., Rodríguez-Iturbe B. (2006). Mechanisms of disease: Oxidative stress and inflammation in the pathogenesis of hypertension. Nat. Clin. Pract. Nephrol..

[82] Jang H.L., Liceaga A.M., Yoon K.Y. (2017). Isolation and characteristics of anti-inflammatory peptides from enzymatic hydrolysates of sandfish (*Arctoscopus japonicus*) protein. J. Aquat. Food Prod. Technol..

[83] Béné C., Barange M., Subasinghe R., Pinstrup-Andersen P., Merino G., Hemre G.-I., Williams M. (2015). Feeding 9 billion by 2050—Putting fish back on the menu. Food Secur..

